# Higher Maternal Diet Quality during Pregnancy and Lactation Is Associated with Lower Infant Weight-For-Length, Body Fat Percent, and Fat Mass in Early Postnatal Life

**DOI:** 10.3390/nu11030632

**Published:** 2019-03-15

**Authors:** Muna J. Tahir, Jacob L. Haapala, Laurie P. Foster, Katy M. Duncan, April M. Teague, Elyse O. Kharbanda, Patricia M. McGovern, Kara M. Whitaker, Kathleen M. Rasmussen, David A. Fields, David R. Jacobs, Lisa J. Harnack, Ellen W. Demerath

**Affiliations:** 1 Division of Epidemiology and Community Health, University of Minnesota, Minneapolis, MN 55454, USA; haap0016@umn.edu (J.L.H.); fost0112@umn.edu (L.P.F.); jacob004@umn.edu (D.R.J.J.); harna001@umn.edu (L.J.H.); ewd@umn.edu (E.W.D.); 2 Department of Pediatrics, University of Oklahoma Health Sciences Center, Oklahoma City, OK 73104, USA; Katy-Duncan@ouhsc.edu (K.M.D.); April-Teague@ouhsc.edu (A.M.T.); David-Fields@ouhsc.edu (D.A.F.); 3 HealthPartners Institute, Minneapolis, MN 55425, USA; elyse.o.kharbanda@healthpartners.com; 4 Division of Environmental Health Sciences, University of Minnesota, Minneapolis, MN 55455, USA; pmcg@umn.edu; 5 Department of Health and Human Physiology, University of Iowa, Iowa City, IA 52242, USA; kara-whitaker@uiowa.edu; 6 Department of Epidemiology, University of Iowa, Iowa City, IA 52246, USA; 7 Division of Nutritional Sciences, Cornell University, Ithaca, NY 14850, USA; kathleen.rasmussen@cornell.edu

**Keywords:** maternal diet quality, pregnancy, lactation, infancy, growth, body composition

## Abstract

Maternal pregnancy nutrition influences fetal growth. Evidence is limited, however, on the relationship of maternal diet during pregnancy and lactation on infant postnatal growth and adiposity. Our purpose was to examine associations between maternal diet quality during pregnancy and lactation with offspring growth and body composition from birth to six months. Maternal diet quality was serially assessed in pregnancy and at one and three months postpartum, using the Healthy Eating Index–2015 in a cohort of 354 fully breastfeeding mother–infant dyads. Infant length-for-age (LAZ), weight-for-age (WAZ), and weight-for-length (WLZ) Z-scores were assessed at birth, one, three, and six months. Infant body fat percent (BF%), fat mass (FM), and fat-free mass (FFM) were measured at six months using dual-energy X-ray absorptiometry. Higher maternal diet quality from pregnancy through three months postpartum was associated with lower infant WLZ from birth to six months (*p* = 0.02) and BF% at six months (*p* ≤ 0.05). Higher maternal diet quality at one and three months postpartum was also associated with lower infant FM at six months (*p* < 0.01). In summary, maternal diet quality during pregnancy and lactation was inversely associated with infant relative weight and adiposity in early postnatal life. Additional research is needed to explore whether associations persist across the life course.

## 1. Introduction

The in utero environment is known to play an important role in fetal programming and subsequent offspring growth and development [1]. Intrauterine nutritional and environmental exposures may have adverse effects on lifelong organ structure and function, which ultimately influence offspring health and disease risk [1,2]. Within this context, research has shown that maternal pre-pregnancy body mass index (BMI) [3], gestational weight gain [3,4], and lifestyle during pregnancy [5] may influence offspring obesity. These effects may appear immediately in the form of macrosomia and birth weight large-for-gestational age, or may be latent, with adiposity appearing later in life [3]. The role of maternal diet during this window of vulnerability is of particular interest for obesity prevention [6]. Although maternal obesity is the strongest predictor of offspring obesity [3], the modifiability of maternal diet is potentially a crucial tool in combating obesity in offspring.

Accruing animal and human evidence also indicates that maternal nutrition beyond the pregnancy period may play a role in early life infant health and subsequent obesity and chronic disease risk [7,8,9]. For example, exclusive breastfeeding is recommended by the American Academy of Pediatrics and other as the optimal nutritional source for infants and is currently initiated by the majority of United States mothers [10,11]. It is possible that maternal diet influences breast milk composition, providing a pathway by which maternal diet may directly influence offspring growth [12]. It is known, for instance, that the composition of breast milk may alter epigenetic programming [13] and the infant gut microbiome and later health and obesity risk [14]. Maternal diet preferences also indirectly influence the general nutritional environment the infant is exposed to in the household through her purchasing and child feeding patterns [15].

Studies exploring the associations of maternal diet during pregnancy and lactation with offspring anthropometry or body composition have often focused on specific dietary components and the macronutrient composition of diet with inconsistent findings [8,16,17,18]. Dietary intake as measured by “single nutrients” is difficult to interpret given the interrelationships of most nutrients and biologically active components within and among foods and the associations of overall food-intake patterns with health [19]. Indices of diet quality, rather than specific nutrient intakes, may better reflect broader food-intake patterns, intakes of combinations of foods, nutrient-nutrient interactions, and may be more generalizable across populations [19]. A number of such indicators of diet quality have been developed and are increasingly used in health outcomes research [20].

There is a paucity of research assessing the relationship of maternal diet quality across the entire perinatal period (including both pregnancy and lactation) with offspring growth and body composition in early life [21,22,23,24,25]. Most studies have focused on anthropometry at birth [21,22] or in childhood [24], with few evaluating repeated measures of growth during infancy, a period of rapid change with potential effects extending across the lifespan [26]. Furthermore, limited research has examined the impact of maternal diet quality during pregnancy and lactation on measures of offspring body composition [23,25], which may provide different insights into growth trajectories and chronic disease risk [27].

The purpose of the present study is to evaluate associations of maternal diet quality during pregnancy and lactation with measures of infant growth (length-for-age (LAZ), weight-for-age (WAZ), and weight-for-length (WLZ) Z-scores from birth to six six months of age) and body composition (body fat percent (BF%)), fat mass (FM), and fat-free mass (FFM) at six months of age). We hypothesized that higher maternal diet quality (as measured by higher Healthy Eating Index–2015 (HEI–2015) total scores) would be associated with higher infant LAZ and lower infant WAZ and WLZ from birth to six months. We further hypothesized that higher maternal diet quality would be related to lower infant BF% and FM and higher infant FFM at six months of age.

## 2. Materials and Methods

### 2.1. Study Population

Mother–infant dyads included in these analyses were enrolled in the Mothers and Infants LinKed for Health (MILK) study, an ongoing prospective cohort study [28]. Mothers and their infants were recruited from Minneapolis, MN, and Oklahoma City, OK. A total of 367 pregnant women aged 21–45 years, with a pre-pregnancy BMI of 18.5–40.0 kg/m^2^, an intention to exclusively breastfeed for at least 3 months, a healthy delivery (defined as <3 days in the hospital for vaginal deliveries and <5 days in the hospital for caesarean section deliveries), and who delivered singleton infants born at-term with a birth weight of ≥2500–≤4500 g were recruited into the study. Exclusion criteria for the study were as follows: (1) mothers consumed tobacco or >1 alcoholic drink per week during pregnancy/lactation; (2) mothers had a history of Type 1 or Type 2 diabetes or current gestational diabetes; (3) mothers had a congenital illness affecting infant feeding or growth; and/or (4) mothers were unable to speak or understand English. The final analytic sample included 354 mother–infant dyads who had maternal dietary data and infant growth measures on at least one of the time points when these items were measured.

The MILK study protocols were approved by the institutional review boards of the University of Minnesota, HealthPartners Institute and the University of Oklahoma Health Sciences Center. All women provided written informed consent at baseline and compensation was provided after each measurement visit.

### 2.2. Maternal Diet

Maternal dietary intake data was collected during the third trimester of pregnancy and at one and three months postpartum using the Diet History Questionnaire II (DHQ II), a food frequency questionnaire (FFQ) designed to assess frequency of intake and portion sizes for 134 food and beverage items and 8 dietary supplement items over the previous month [29]. A prior version of the DHQ II (DHQ I) with minimal modifications to food lists and nutrient databases has been evaluated for validity in prior research [29,30,31]. While one study showed that the DHQ I performed better than the Willett FFQ and Block FFQ at estimating energy and absolute nutrient intake [29], others showed that men and women under-reported energy and protein intakes on the DHQ I [30] which may lead to attenuation of estimated disease relative risks for these nutrients [31]. The DHQ II was analyzed using Diet Calc (National Cancer Institute, Bethesda, MD, USA), and food and nutrient values were generated using the United States Department of Agriculture (USDA) Food Patterns Equivalent Database and the Food and Nutrient Database for Dietary Studies.

Maternal diet quality during pregnancy and lactation was estimated using the HEI–2015, a scoring system designed to measure adherence to the 2015–2020 Dietary Guidelines for Americans (2015–2020 DGA) [32]. The psychometric properties of the HEI–2015 were evaluated using data from exemplary menus, a nationally representative sample and a prospective cohort study, with evidence supporting construct validity and reliability and criterion validity [33]. Previous studies have assessed the validity of prior HEI versions among pregnant women and showed that the index was useful in providing a composite dietary intake measure [34].

The HEI–2015 total score is the sum of 13 subcomponent scores that measure adequacy (Total Fruits, Whole Fruits, Total Vegetables, Greens and Beans, Whole Grains, Dairy Products, Total Protein Foods, Seafood and Plant Proteins, and Unsaturated:Saturated fats) and moderation (Refined Grains, Sodium, Added Sugars, and Saturated Fats). All subcomponents are scored from 0–5 or 0–10 based on intake between the minimum and maximum standards. Moderation components are reverse scored such that higher scores reflect lower intake. To account for variation in caloric intake between participants, all HEI–2015 components are standardized to 1000 kilocalories, except for the ratio of unsaturated to saturated fats. A higher HEI–2015 total score (out of 100) represents greater consistency of the diet with the 2015–2020 DGA (32). The code used to calculate the HEI–2015 scores was developed by the Division of Cancer Control and Population Sciences in the National Cancer Institute [35].

### 2.3. Infant Growth and Body Composition

At birth, one, three, and six months, infant length was measured with the infant undressed, but diapered using the Seca 416 infantometer (Seca, Birmingham, UK). Infant naked weight was measured using the high sensitivity scale embedded in the PEA POD (COSMED USA, Inc., Concord, CA, USA). Age and sex-specific LAZ, WAZ, and WLZ were subsequently calculated using the World Health Organization Z-score classification system for term infants [36]. At six months, infant BF%, FM, and FFM were assessed utilizing dual X-ray energy absorptiometry (DXA), with the infant in a supine position wearing only a disposable diaper and swaddled in a light cotton blanket [37]. All measurements were obtained using standardized protocols and after cross-training of study staff [38].

### 2.4. Statistical Analyses

To provide a comprehensive description of the mother–infant dyads, demographic and clinical characteristics are presented as means ± standard deviations (SD) for continuous variables, and frequencies for categorical variables by tertiles of maternal HEI–2015 total scores during pregnancy. Differences in participant characteristics by maternal diet quality during pregnancy were evaluated using one-way ANOVAs and chi-square tests for continuous and categorical variables, respectively. Differences in participant characteristics among dyads who had complete dietary data during pregnancy and infant WLZ at birth (*N* = 319) versus those who had complete dietary data at three months postpartum and infant WLZ at six months (*N* = 257) were compared using *t*-tests and chi-square tests for continuous and categorical variables, respectively. Pearson correlations were calculated to test correlations between maternal HEI–2015 total scores during pregnancy and at one and three months postpartum.

Linear mixed effects models (PROC MIXED) were then used to evaluate the associations of repeated measures of maternal diet quality (HEI–2015 total scores) from pregnancy through three months postpartum with within-subject measures of infant LAZ, WAZ, and WLZ from birth to six months. The exposures were analyzed as continuous variables owing to the relatively limited sample size and after studying goodness of linear fit using scatter plots. The data were first adjusted for study site (Minneapolis, Oklahoma), maternal age (years), race (white, other), education (high school/GED/associates degree, Bachelor’s degree, graduate degree), household income (<$60,000, $60,000–90,000, >$90,000), and total energy intake (time-dependent; kilocalories) (Model 1). The full models (Model 2) were additionally adjusted for the following potential confounders: pre-pregnancy BMI (kg/m^2^), gestational weight gain (kilograms), parity (0, ≥1), mode of delivery (vaginal, cesarean section), duration of exclusive breastfeeding (one, three, six months), infant gestational age (weeks), and infant sex (male, female). The main exposures and all confounders were included as main effects, but interactions with time were only retained if the corresponding *p*-value estimates were significant at <0.05.

Next, linear regression models (PROC GLM) were run to examine separately the associations of maternal diet quality during pregnancy, one and three months postpartum with infant BF%, FM, and FFM at six months of age. The models were similarly adjusted for all aforementioned potential confounders (Models 1 and 2), with the addition of infant WLZ at birth and infant exact age at six months (Model 2). Given that infant fat mass index (FMI) and fat-free mass index (FFMI) scale to length (fat mass or fat-free mass/length^2^) and have been proposed as more precise indicators of adiposity and nutrition status [39], we also tested the associations of maternal perinatal diet quality with these indices. We further explored associations of maternal diet during pregnancy and lactation with compartment specific fat mass (trunk, arm, and leg) to detect changes in the relative distribution of fat within the body in sub-analyses.

Lastly, in sensitivity analyses to examine if the relationship of maternal diet with infant growth and/or body composition is modified by exclusivity of breastfeeding, the full models for both infant growth and body composition were re-run to include only infants who were fully breastfed to six months. All statistical analyses were performed using SAS 9.4 (SAS Institute Inc., Cary, NC, USA).

## 3. Results

### 3.1. Participant Characteristics

The mothers included in the present study were mostly highly educated, white, multiparous, fully breastfeeding at six months with average gestational weight gain, and above average HEI–2015 scores during pregnancy and lactation (Table 1). Maternal education, parity, HEI–2015 scores at one and three months postpartum, and infant gestational age differed significantly by tertiles of maternal HEI–2015 scores during the third trimester of pregnancy, whereby mothers with higher pregnancy HEI–2015 scores were on average more educated, nulliparous, had higher postpartum HEI–2015 scores and delivered their infants later (*p* < 0.05). No significant differences were observed between characteristics of the mother–infant dyads who had complete dietary data during pregnancy/infant WLZ at birth and complete dietary data at three months postpartum/infant WLZ at six months postpartum (*p* > 0.05; data not shown). Maternal HEI–2015 scores during pregnancy were correlated with scores at one and three months postpartum (*r* = 0.60 and *r* = 0.65; *p* < 0.001, respectively). HEI–2015 scores at one and three months postpartum were also correlated with one another (*r* = 0.63, *p* < 0.001).

As expected, higher intake of all adequacy components (except Dairy Products) and lower intake of all moderation components were seen among mothers in the highest versus lowest tertile of HEI–2015 total scores (Supplementary file Table S1). Higher (better) scores for Whole and Total Fruits, Total Vegetables, Greens and Beans, Seafood and Plant Proteins, Total Protein Foods, Refined Grains, and Added Sugars appeared to drive higher average HEI–2015 total scores. HEI–2015 total scores were normally distributed at all time points and average scores were highest during the third trimester of pregnancy (Supplementary file Figure S1).

Among infants, on average, LAZ decreased from birth to six months, whereas WAZ and WLZ decreased from birth to three months, followed by a subsequent increase after three months (Table 2). The mean fat mass among infants at six months was approximately 2.8 kg (34% of total body weight), with most of the fat distributed in the legs (1.08 kg) and trunk (0.92 kg).

### 3.2. Maternal Diet Quality and Infant Growth

Maternal diet quality through pregnancy and lactation was inversely and significantly associated with WLZ, such that a 10-unit increase in the HEI–2015 total score from pregnancy through three months postpartum was associated with an approximately 0.12 lower infant WLZ from birth to six months in the fully-adjusted model (β = −0.12, *p* = 0.02) (Table 3). There was no significant interaction between maternal diet quality from pregnancy through three months postpartum and time, suggesting no difference between prenatal and postnatal exposure on these outcomes. No associations of maternal diet quality from pregnancy through three months postpartum with infant LAZ or WAZ were observed. In sensitivity analyses, findings for infant LAZ, WAZ, and WLZ were comparable when models were restricted to the 75% of study infants who were fully breastfed to six months.

### 3.3. Maternal Diet Quality and Infant Body Composition

Maternal diet quality during pregnancy and lactation was also inversely associated with infant adiposity (Table 4). Specifically, a 10-unit increase in HEI–2015 total scores during pregnancy was associated with an approximately 0.6% lower infant BF% at six months (β = −0.58, *p* = 0.05). A 10-unit increase in HEI–2015 total score at one month postpartum was associated with more than a 1% lower infant BF% (β = −1.28, *p* < 0.001) and 0.10 kg lower FM (β = −0.13, *p* = 0.001) at six months. Likewise, a 10-unit increase in HEI–2015 total score at three months postpartum was associated with an approximately 0.7% lower infant BF% (β = −0.66, *p* = 0.01) and 0.10 kg lower FM (β = −0.10, *p* = 0.01) at six months. Findings were similar for associations between maternal diet quality at one and three months postpartum and FMI/FFMI (data not shown). In sub-analyses, a 10-unit increase in HEI–2015 total scores at one and three months postpartum were associated with both lower trunk FM (β = −0.06, *p* = 0.001 and β = −0.04, *p* = 0.03, respectively) and lower leg FM (β = −0.05, *p* = 0.01 and β = −0.04, *p* = 0.03, respectively). No other associations between maternal HEI–2015 scores during pregnancy or lactation and infant body composition were found. In sensitivity analyses that restricted the analysis to study infants that were fully breastfeeding at six months of age, associations between maternal HEI–2015 scores and infant body composition were similar to those incorporating all infants, except for associations between maternal HEI–2015 scores at three months postpartum and infant BF% and FM at six months, which were no longer significant (*p* < 0.10).

## 4. Discussion

In this prospective cohort study, we examined the associations of maternal diet quality during pregnancy and lactation with infant growth from birth to six months and body composition at six months. We found that higher maternal diet quality (as evidenced by higher HEI–2015 scores) from pregnancy through three months postpartum was inversely associated with infant WLZ from birth to six months and BF% at six months. Similarly, higher maternal diet quality during lactation (one and three months postpartum) was inversely related to infant FM (specifically trunk and leg fat mass) at six months. These associations occurred independently of important confounders, such as pre-pregnancy BMI and gestational weight gain. This research adds to accumulating animal and human evidence that suggests that not only the in utero but also the postnatal nutritional environment influences infant growth and body composition in infants who are breastfed and points to a potentially important role of dietary constituents and quality in shaping offspring future health [6].

Our findings are concordant with those of several other studies assessing mostly maternal prenatal diet quality and offspring growth and/or body composition at birth or later in life [21,22,23,24,25]. In line with our findings, Shapiro et al. [23] showed that maternal HEI–2010 scores of ≤57 (v > 57) measured via repeated 24-h recalls throughout pregnancy were associated with higher infant BF% (β = 0.58, *p* < 0.05) and FM (β = 20.74, *p* < 0.05) at birth among mother-offspring pairs in the Healthy Start Study in the United States. Likewise, in a pooled analysis of mother-child pairs from Project Viva (United States) and the Rhea study (Greece) [24], a three-point increase in the Mediterranean diet score during the first—second trimester of pregnancy (evaluated using an FFQ) was associated with lower offspring BMI Z-scores in mid-childhood at a magnitude similar to that found for WLZ in our study (β = −0.14, 95% CI = −0.15, −0.13).

Conversely, several studies have not found significant associations between maternal prenatal diet and infant growth [8,21,40]. For example, using data from the Infant Feeding Practices Study II, Poon et al. [21] showed that both the alternate Mediterranean diet score and the Alternative Healthy Eating Index for Pregnancy (AHEI-P) diet score (measured using an FFQ during the third trimester of pregnancy) were unrelated to infant birth weight, birth size (large or small for gestational age), birth WLZ or change in WLZ at 4–6 months. Similarly, Rifas-Shiman et al. [40] reported a non-significant lower risk of giving birth to a small-for-gestational age infant with increasing AHEI-P scores calculated from responses to an FFQ during the first trimester of pregnancy among mother–infant dyads in Project Viva. The literature does not adequately assess the role of maternal postpartum diet on infant growth and/or body composition, which theoretically influences the infant via breast milk or the household nutritional environment. In one study assessing maternal macronutrient intake during the first trimester of pregnancy and at five years postpartum, Murrin et al. [8] demonstrated that higher prenatal sugar intake and both prenatal and postnatal saturated fat intake as measured by an FFQ were associated with a non-significant trend towards higher likelihood of having overweight/obese offspring. Although many diet quality scores were moderately to strongly correlated in various populations [41,42], use of the HEI–2015 as a measure of diet quality during pregnancy and postpartum may explain some discrepancies in findings and may limit our ability to compare findings to other studies using different diet quality scores or prior versions of the HEI.

The HEI–2015 aims to reflect the most recent DGA (2015–2020) and is thus generalizable to other studies using these guidelines [32]. The mean HEI–2015 scores across pregnancy and lactation ranged from 65.9 to 67.2 in our cohort. These scores are higher than the average scores for American adults (~58 out of 100) [43] but may reflect the potential improvement in dietary intake that women may pursue during the perinatal period [44]. Our study population is also more socioeconomically advantaged and healthy relative to pregnant and lactating women in the United States, as evidenced by a higher maternal education level, household income, lower pre-pregnancy BMI, and longer duration of exclusive breastfeeding. Given the dearth of studies that parallel the timing and methods of our exposure and outcome assessment and confounders considered, additional research is warranted to replicate our findings in larger, more diverse cohorts.

It is important to note that, although our findings are qualitatively similar to those of several other studies of maternal perinatal diet with infant growth and/or body composition [23,24], our effect sizes and those of related studies are small relative to the contribution of other maternal factors, such as obesity and gestational weight gain [45]. Even so, it is likely that infant adiposity is multifactorial, with several elements each contributing small effects to overall growth and fat accrual [46]. Recognizing and targeting these immediately modifiable factors, such as maternal diet in holistic interventions, could have a significant influence on offspring obesity throughout the lifespan [47]. For example, within the context of our study, a 10-unit increase in the HEI–2015 score could translate into increasing consumption of fruits from none to ≥0.8 cup equivalents to maximize points for this adequacy subcomponent (10 points). Alternatively, mothers could reduce consumption of refined grains from ≥4.3 oz equivalents to ≤1.8 oz equivalents to maximize points for this moderation subcomponent (10 points).

Maternal perinatal diet quality may impact infant growth and/or body composition through numerous mechanisms. Aside from direct influences on fetal growth, maternal prenatal and postnatal nutrition may lead to epigenetic modifications that affect offspring metabolic function, growth hormone secretion and appetite programming [13,48]. Higher HEI scores were associated with greater intake of fruits, fibers, folate, and vitamin C, higher plasma concentrations of carotenoids and vitamin C, and greater dietary variety in previous research [49]. In our study, we found that mothers in the highest tertile of HEI–2015 had especially high scores (scores that were greater than or equal to 80% of the maximum possible points for index components) on the Whole and Total Fruits, Total Vegetables, Greens and Beans, Seafood and Plant Proteins, Total Protein Foods, Refined Grains, and Added Sugars components. These beneficial characteristics of maternal diet are associated with reduced oxidative stress and inflammation, which may be reflected in the nutritional environment in the household during this critical period of offspring growth and development and throughout the life course [50]. Maternal diet may also alter the fatty acid profile [51] and the hormonal [52] and growth factor [53] content of breast milk the infants in this study were ingesting, which could theoretically influence growth and body composition [54].

Our study has several notable strengths, including the prospective cohort design with repeated measures of maternal diet from pregnancy through postpartum and infant growth from birth to six months. These factors provide a valuable opportunity to assess concurrent and prospective associations of maternal nutrition with offspring growth at a critical period of sensitivity in the life course. Our use of overall diet quality as an exposure (*v* specific macronutrients) may better mirror whole diet, accounting for the synergistic, interactive, and cumulative effects of multiple foods and nutrients, and may be a more practical tool for nutrition communication throughout pregnancy and lactation [19]. We also measured infant body composition at six months of age using DXA, which accurately measures fat mass and soft-tissue body components and serves as a marker of potential developmental programming [55]. We were able to account for the influence of a range of demographic, clinical, and lifestyle factors, which may confound the association of maternal diet and offspring growth and/or body composition.

We acknowledge potential limitations in our study. Maternal dietary intake was self-reported by mothers using the DHQ-II, which is subject to recall bias, measurement error, and exposure misclassification [56,57]. Nonetheless, this FFQ has been evaluated for validity in adult populations [29] and our models adjusted for total energy intake to reduce potential systematic measurement error. Our relatively small sample size and loss of data at later time points may have decreased statistical power. However, mother–infant dyads with complete exposure and outcome data during pregnancy/at birth were similar to those with complete exposure and outcome data at three months postpartum/six months. Our study population comprises predominantly non-Hispanic white women, which may limit generalizability across race/ethnicities. We did not follow-up infants beyond six months of age, which may limit our understanding of the long-term consequences of maternal nutrition on offspring growth, body composition and cardiometabolic diseases across the life course [58,59]. Important confounding variables may have not been controlled for in analyses. Most notably, it is possible that maternal diet is a marker for infant feeding practices that may influence child growth outcomes (including responsive infant feeding and timing of introduction of solid foods). Lastly, given the observational nature of the study, it is not possible to draw causal inferences from observed associations.

## 5. Conclusions

In conclusion, we found that higher maternal diet quality from pregnancy through three months postpartum was associated with lower infant WLZ from birth to six months and lower infant BF% at six months. Likewise, higher maternal diet quality at one and three months postpartum was associated with lower total body, trunk, and leg FM at six months. These findings point to the importance of focusing on maternal diet quality at critical periods of development, and the appreciable, albeit small, independent influence that maternal nutrition may play in optimal infant growth and fat development. Additional research is needed to determine the interplay between maternal perinatal diet quality, long-term offspring growth/body composition, and later chronic disease risk susceptibility in hopes of aiding current efforts to develop more specific dietary guidelines for pregnant and postpartum women.

## Figures and Tables

**Table 1:** Participant demographic and clinical characteristics by tertiles of maternal HEI–2015 total scores during the third trimester of pregnancy (*n* = 329).

Participant Characteristics		HEI–2015 Tertiles (T) ^a^			
	Total	T1 (*n* = 109)	T2 (*n* = 110)	T3 (*n* = 110)	
	N (%) or Mean ± SD				*p*-Value
Study site					0.47
Minnesota	233 (66)	69 (63)	72 (65)	78 (71)	
Oklahoma	121 (34)	40 (37)	38 (35)	32 (29)	
Age, years	30.9 ± 4.1	30.2 ± 4.3	30.7 ± 4.4	31.4 ± 3.7	0.1
Race					0.71
White	299 (86)	92 (85)	93 (87)	97 (89)	
Other	49 (14)	16 (15)	14 (13)	12 (11)	
Education					<0.001 *
High school/GED/Associates degree	80 (24)	37 (36)	26 (24)	11 (11)	
Bachelor’s degree	136 (40)	42 (41)	38 (36)	48 (46)	
Graduate degree	121 (36)	24 (23)	42 (40)	45 (43)	
Household income					0.19
<$60,000	105 (31)	37 (36)	34 (32)	25 (24)	
$60,000–90,000	85 (25)	30 (29)	24 (23)	27 (26)	
>90,000	147 (44)	36 (35)	48 (45)	52 (50)	
Parity					0.01 *
0	146 (42)	33 (31)	54 (50)	52 (47)	
≥1	204 (58)	74 (69)	55 (50)	58 (53)	
Pre-pregnancy BMI, kg/m^2^	26.4 ± 5.4	27.2 ± 6.2	26.4 ± 4.9	25.5 ± 5.0	0.06
Gestational weight gain					0.23
Below or within IOM guidelines	200 (57)	54 (501)	67 (62)	63 (57)	
Exceeding IOM guidelines	149 (43)	53 (50)	41 (38)	47 (43)	
Energy intake during pregnancy	1827 ± 530	1747 ± 550	1876 ± 535	1859 ± 498	0.15
Mode of delivery					0.39
Vaginal	278 (80)	90 (83)	82 (76)	87 (81)	
Caesarean section	70 (20)	18 (17)	26 (24)	21 (19)	
HEI–2015 score during third trimester of pregnancy	67.2 ± 8.7	57.5 ± 5.8	67.8 ± 2.1	76.1 ± 4.0	<0.001 *
HEI–2015 score at one month postpartum	65.9 ± 8.4	60.6 ± 7.5	65.2 ± 7.0	71.2 ± 7.6	<0.001 *
HEI–2015 score at three months postpartum	66.1 ± 8.7	60.5 ± 7.9	65.3 ± 7.2	71.5 ± 7.4	<0.001 *
Duration of exclusive breastfeeding					
one month	21 (7)	7 (7)	10 (10)	2 (2)	0.16
three months	58 (18)	20 (22)	16 (16)	20 (19)	
six months	235 (75)	65 (71)	74 (74)	81 (79)	
Infant gestational age, weeks	39.8 ± 1.1	39.7 ± 1.0	39.6 ± 1.1	40.0 ± 1.1	0.04 *
Infant sex					0.84
Male	178 (50)	53 (49)	57 (52)	53 (48)	
Female	176 (50)	56 (51)	53 (48)	57 (52)	

Abbreviations: HEI = Healthy Eating Index; BMI = Body Mass Index; IOM = Institute of Medicine. ^a^ HEI–2015 T1: ≤63.9; T2: 64.0–70.9; T3: ≥70.9. * *p* < 0.05 for tests of differences in participant characteristics by tertiles of maternal HEI–2015 total scores during pregnancy using chi-square and one-way ANOVAs for categorical and continuous variables, respectively. Data are presented as mean ± SD or column percentages.

**Table 2:** Average infant growth measures from birth to six months and body composition measures at six months.

Infant Characteristics	N	Mean ± SD
Infant Growth from Birth to Six Months		
Weight-for-age, Z-scores		
Birth	350	0.46 ± 0.87
one month	343	0.13 ± 0.87
three months	332	−0.07 ± 0.88
six months	321	0.04 ± 0.96
Length-for-age, Z-scores		
Birth	342	1.17 ± 1.23
one month	346	0.06 ± 1.09
three months	332	−0.03 ± 1.06
six months	321	−0.20 ± 1.09
Weight-for-length, Z-scores		
Birth	341	−0.72 ± 1.40
one month	342	0.12 ± 1.08
three months	332	0.02 ± 0.98
six months	321	0.33 ± 1.07
Infant body composition at six months		
Total body fat, %	317	33.98 ± 3.76
Fat mass, kg	317	2.76 ± 0.48
Fat-free mass, kg	317	5.29 ± 0.67
Trunk fat mass, kg	317	0.92 ± 0.22
Arm fat mass, kg	317	0.38 ± 0.20
Leg fat mass, kg	317	1.08 ± 0.23

Abbreviations: SD = standard deviation.

**Table 3:** Associations of HEI–2015 total scores from the third trimester of pregnancy through three months postpartum with infant growth measures from birth to six months.

	Model 1				Model 2			
Infant Growth Measures from Birth to Six Months	N	β ^a^	SE	*p*-Value	N	β ^a^	SE	*p*-Value
LAZ	330	0.05	0.04	0.25	290	0.02	0.04	0.58
WAZ	330	−0.02	0.03	0.43	290	−0.04	0.03	0.15
WLZ	330	−0.13	0.05	**0.01**	290	−0.12	0.05	**0.02**

Abbreviations: SE = standard error; LAZ = length-for-age Z-score; WAZ = weight-for-age Z-score; WLZ = weight-for-length Z-score. ^a^ HEI–2015 total score was converted such that a 1-unit increase corresponds to a 10-point increase in HEI–2015 score. Model 1 = adjusted for study site, maternal age, race, education, household income and total energy intake during pregnancy. Model 2 = Model 1 + maternal pre-pregnancy BMI, gestational weight gain, mode of delivery, parity, breastfeeding exclusivity at six months, infant sex, and gestational age. Bolded values are statistically significant at *p* < 0.05.

**Table 4:** Associations of HEI–2015 total scores during the third trimester of pregnancy and at one and three months postpartum with infant body composition at six months.

	Model 1				Model 2			
Infant Body Composition Measures at Six Months	N	β ^a^	SE	*p*-Value	N	β ^a^	SE	*p*-Value
HEI–2015 total scores during pregnancy								
BF%	281	−0.72	0.28	**0.01**	262	−0.58	0.29	**0.05**
FM	281	−0.06	0.04	0.12	262	−0.03	0.04	0.36
FFM	281	0.002	0.05	0.97	262	−0.001	0.05	0.99
HEI–2015 total scores at one month postpartum								
BF%	254	−1.22	0.30	**<0.001**	235	−1.28	0.30	**<0.001**
FM	254	−0.10	0.04	**0.01**	235	−0.13	0.04	**0.001**
FFM	254	−0.003	0.05	0.94	235	−0.05	0.04	0.28
HEI–2015 total scores at three months postpartum								
BF%	248	−0.69	0.25	**0.01**	229	−0.66	0.26	**0.01**
FM	248	−0.09	0.04	**0.02**	229	−0.10	0.04	**0.01**
FFM	248	−0.04	0.05	0.52	229	−0.05	0.06	0.37

Abbreviations: BF% = body fat percent; FM = fat mass; FFM = fat free mass. ^a^ HEI–2015 total score was converted such that a 1-unit increase corresponds to a 10-point increase in HEI–2015 score. Model 1 = adjusted for study site, maternal age, race, education, household income, and total energy intake during pregnancy. Model 2 = Model 1 + maternal pre-pregnancy BMI, gestational weight gain, mode of delivery, parity, breastfeeding exclusivity at six months, infant sex, gestational age, weight-for-length Z-score, and exact age at body composition assessment. Bolded values are statistically significant at *p* < 0.05.

## Supplementary Materials

The following are available online at https://www.mdpi.com/2072-6643/11/3/632/s1, Table S1: Maternal HEI–2015 subcomponent scores and energy intake during pregnancy by tertiles of maternal HEI–2015 total scores during pregnancy (*n* = 329); Figure S1: Distribution of Maternal Healthy Eating Index-2015 (HEI–2015) scores: (a) during the third trimester of pregnancy (T1: ≤63.9; T2: 64.0–70.9; T3: ≥70.9); (b) at one month postpartum (T1: ≤61.8; T2: 61.9–69.7; T3: ≥69.8); and (c) at three months postpartum (T1: ≤62.8; T2: 62.9–70.2; T3: ≥70.3).
