# Supplementary material for: Higher Maternal Diet Quality during Pregnancy and Lactation Is Associated with Lower Infant Weight-For-Length, Body Fat Percent, and Fat Mass in Early Postnatal Life
Source: Nutrients. 2019 Mar 15;11(3):632. doi: 10.3390/nu11030632 (PMC6471184; doi:10.3390/nu11030632)
Supplement: Supplementary file 1 [file nutrients-11-00632-s001.pdf]

nutrients

Table S1. Maternal Healthy Eating Index–2015 (HEI–2015) subcomponent scores during pregnancy by tertiles of maternal HEI–2015 total scores during pregnancy (n=329).

	HEI–2015 Tertiles (T)a			
	T1 (n=106)	T2 (n=107)	T3 (n=106)	
HEI–2015 subcomponent scores		Mean ± SD		P-value
Total Fruits[b]	3.8 ± 1.4	4.6 ± 0.8	4.9 ± 0.3	<0.001*
Whole Fruits[b]	4.4 ± 1.1	5.0 ± 0.2	5.0 ± 0.0	<0.001*
Total Vegetables[b]	3.4 ± 1.0	3.9 ± 0.9	4.0 ± 0.9	<0.001*
Greens and Beans[b]	2.9 ± 1.5	4.3 ± 1.2	4.5 ± 1.0	<0.001*
Whole Grains[c]	2.2 ± 1.1	2.8 ± 1.4	3.4 ± 1.7	<0.001*
Refined Grains[c, d]	7.2 ± 2.1	8.2 ± 1.7	9.1 ± 1.2	<0.001*
Dairy Products[c]	7.4 ± 2.3	7.3 ± 2.6	7.3 ± 2.5	0.97
Total Protein Foods[b]	4.4 ± 0.9	4.7 ± 0.7	4.9 ± 0.4	<0.001*
Seafood and Plant Proteins[b]	3.5 ± 1.4	4.4 ± 1.0	4.9 ± 0.4	<0.001*
Unsaturated:Saturated Fats[c]	2.9 ± 1.9	4.7 ± 2.1	7.0 ± 2.3	<0.001*
Sodium[c, d]	4.8 ± 2.3	5.2 ± 1.8	5.9 ± 2.0	<0.001*
Saturated Fats[c, d]	3.3 ± 2.4	4.9 ± 2.1	6.4 ± 1.8	<0.001*
Added Sugars[c, d]	7.3 ± 2.4	8.0 ± 1.9	8.9 ± 1.2	<0.001*

[a]HEI-2015 T1: ≤63.9; T2: 64.0-70.9; T3: ≥70.9
[b]Maximum of 5 points
[c]Maximum of 10 points
[d]Higher scores correspond to lower intake
*P<0.05 for tests of differences in maternal diet composition by tertiles of HEI–2015 total scores during pregnancy using one-way ANOVA

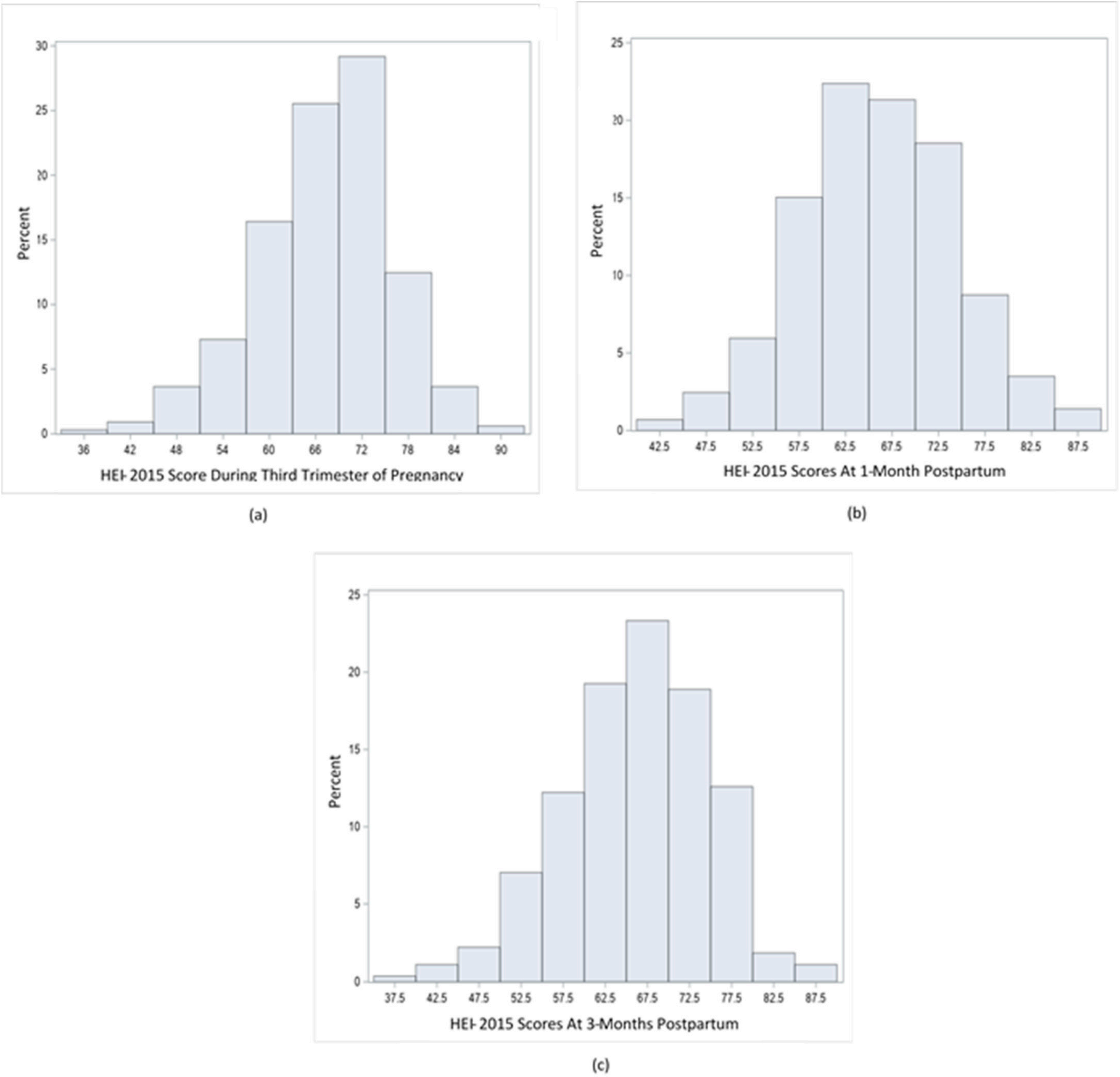

Figure S1. Distribution of Maternal Healthy Eating Index–2015 (HEI–2015) scores: (a) During the third trimester of pregnancy (T1: ≤63.9; T2: 64.0-70.9; T3: ≥70.9); (b) at one month postpartum (T1: ≤61.8; T2: 61.9-69.7; T3: ≥69.8); and (c) at three months postpartum (T1: ≤62.8; T2: 62.9-70.2; T3: ≥70.3). Abbreviations: T1 = tertile 1; T2 = tertile 2; T3 = tertile 3.
